# Does Body Contouring After Bariatric Weight Loss Enhance Quality of Life? A Systematic Review of QOL Studies

**DOI:** 10.1007/s11695-018-3323-8

**Published:** 2018-08-02

**Authors:** Tania Toma, Leanne Harling, Thanos Athanasiou, Ara Darzi, Hutan Ashrafian

**Affiliations:** The Department of Surgery and Cancer, Imperial College London, 10th Floor, Queen Elizabeth the Queen Mother (QEQM) Building, St Mary’s Hospital Campus, Praed Street, W2 1NY London, UK

**Keywords:** Bariatric, Body contouring, Plastic surgery, Cosmetic, Quality of life

## Abstract

Massive weight loss following bariatric surgery can result in excess tissue, manifesting as large areas of redundant skin that can be managed by body contouring surgery. This study aims to quantify the effects of body contouring surgery on indicators of quality of life in post-bariatric patients. A systematic review and meta-analysis of the literature revealed on indices of quality of life in post-bariatric patients, before and after body contouring surgery. Body contouring surgery resulted in statistically significant improvements in physical functioning, psychological wellbeing and social functioning, as well as a reduction in BMI. Body contouring surgery offers a strategy to improve quality of life in patients suffering from the functional and psychosocial consequences of excess skin after bariatric surgery.

## Introduction

Bariatric and metabolic surgery (BS) achieves significant improvements in the multiple comorbidities associated with obesity such as diabetes mellitus, hypertension, hyperlipidaemia, obstructive sleep apnoea and cardiovascular disease [1–4] that in turn result in improvements to quality of life (QOL) [5]. However, these beneficial outcomes in QOL are not always observed. In approximately one third of patients undergoing BS, the adipocutaneous tissue following massive weight loss (MWL) fails to contract, resulting in loose, hanging excess skin [6]. These cutaneous deformities manifest as significant cosmetic and functional impairments that interfere with mobility and activities of daily living. In addition, patients are predisposed to skin infection, skin rashes and dependent lymphoedema [7]. The literature demonstrates that these complications following MWL negatively impact upon QOL and other markers of psychosocial distress, including social isolation, self-esteem and perceptions of body image [8–11].

Unfortunately, these skin deformities following MWL cannot be resolved by modifications in lifestyle, diet and exercise. Post-bariatric body contouring surgery (BCS) offers a solution by surgically removing excess adipose tissue [6]. However, BCS is not routinely offered and there is ongoing debate as to whether BCS is an essential procedure following BS [12]. Much of this debate stems from whether BCS contributes to more than purely cosmetic outcomes. Evidence in the literature to suggest BCS following BS may improve QOL is currently mixed. Early studies reveal that BCS in post-bariatric patients does not result in significant improvements in QOL compared to patients without BCS [13]. However, more recent work suggests that reconstructive surgery following MWL leads to demonstrable improvements in both functional and psychosocial markers of QOL such as ambulation, self-esteem, sexual function and body image [14–18]. The objective of this study was therefore to identify the importance of BCS following BS, through a systematic review and meta-analysis of studies assessing the use of BCS in post-bariatric patients to improve physical, mental and social QOL.

## Methods

### Search Strategy

**Fig. 1:**
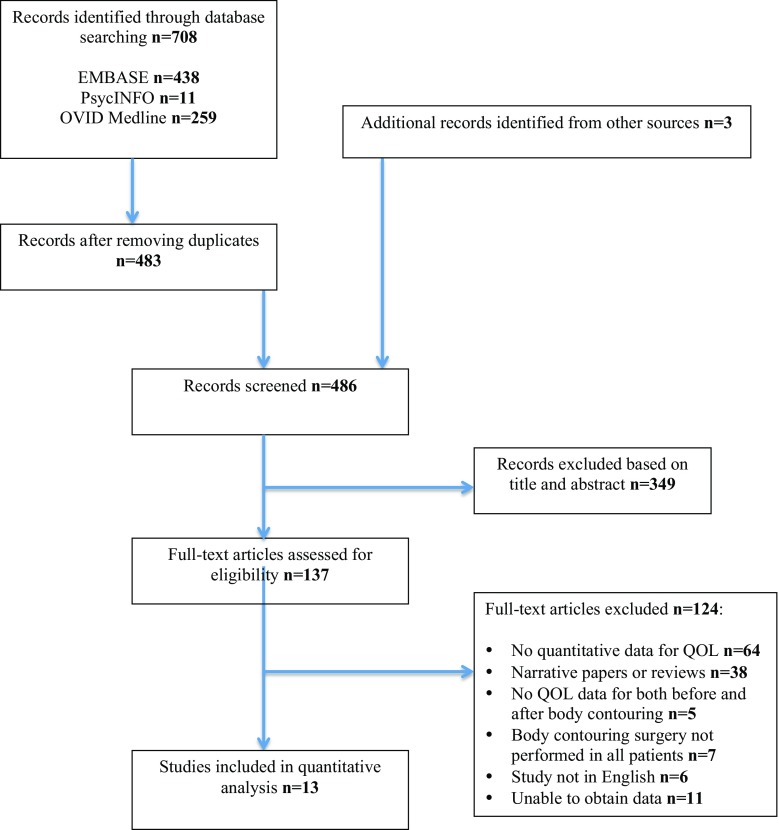
Search strategy according to PRISMA guidelines

Studies published in English were identified by searching EMBASE (1974–August 2017), MEDLINE (1946–August 2017) and PYSCHINFO (1967–August 2017). Combinations of the following search terms were used: ‘bariatric surgery’, ‘body contouring’, ‘plastic surgery’, ‘reconstructive surgery’, ‘quality of life’, ‘body image’, ‘psychosocial function’, ‘psychological function’. Reference lists of identified studies were also searched for the inclusion of additional publications. Our search strategy is summarised in Fig. 1.

### Selection Criteria

Studies were included in analysis if they met the following inclusion criteria: (1) participants had achieved MWL through weight loss surgery prior to BCS, (2) participants underwent one or more body contouring procedures (abdominoplasty, panniculectomy, brachioplasty, thigh lift, etc.), (3) trials measured QOL and/or psychosocial function before and after BCS using a clearly defined instrument.

Studies were excluded if (1) they were editorials, case reports, comments or reviews, (2) data was duplicated, (3) not all participants had previous BS and used other methods to achieve MWL, e.g. lifestyle modification, and (4) they did not use quantitative measures to assess QOL or psychosocial function.

Two reviewers independently assessed the eligibility of studies by reviewing titles and abstracts (TT, HA). If the inclusion criteria were met, full-text articles of eligible studies were obtained for subsequent evaluation.

### Data Extraction and Analysis

The following data from included studies was extracted by two authors independently (TT, HA): first author, journal, year of publication, study design, age and gender of patients, numbers of participants, type of weight loss surgery performed, type of BCS performed, BMI before BS, BMI post-BS, BMI post-BC, instrument used to measure QOL or psychosocial functioning, length of follow-up post-BCS. Our primary outcome was QOL and/or psychosocial functioning before and after BCS in post-bariatric patients.

Each study was assessed and analysed according to clustered common QOL variables, which we identified as (1) physical functioning, (2) psychological wellbeing and anxiety, (3) social consciousness, (4) body image and physical appearance, (5) sexual function, (6) vitality, (7) work ability, (8) pain, (9) self-esteem and (10) global QOL score. Data for each of these variables was extracted pre-BCS and post-BCS and a percentage improvement was calculated.

Meta-analysis was performed in accordance with the Cochrane collaboration and Preferred Reporting Items for Systematic Reviews and Meta-Analyses (PRISMA) guidelines and meta-analysis of observational studies in epidemiology (MOOSE) guidelines [19, 20].

### Quality Scoring

An adapted version of the Newcastle-Ottawa Scale [21] was used to perform quality assessment of included studies. Studies were assessed in three domains: selection of the treatment group, comparability of the treatment groups and assessment of outcomes. Studies scoring at least 5 (out of a maximum of 9) were considered to be of moderate to high quality and were included in subgroup analysis.

Risk of bias was assessed using the Cochrane risk of bias in non-randomised studies (ROBINS-I) [22]. Seven key categories were assessed including confounding, selection, classification of intervention, deviation from intervention, missing data, measurement of outcomes and reporting of results. Risk of bias in each domain was scored as low, moderate, serious or critical. Studies with insufficient information to judge the risk of bias were marked as having ‘no information’. The most serious risk of bias scored in any domain was used to give the overall risk of bias for each study. For example, if a study had a serious risk of bias in any one domain, it would score a serious risk of bias overall despite scores in other domains.

### Statistical Analysis

Quantitative analyses were performed based on controls versus BCS after bariatric surgery. Overall and specific categories of QOL outcomes were analysed by calculating the ratio of means within each study. We substituted median for mean in studies where only the median was reported. The inverse-variance, random effects model of DerSimonian and Laird was used for both continuous and categorical variables. This was accomplished using Stata 13 (StataCorp., College Station, TX, US). The *I*^2^ statistic was used to estimate the degree of heterogeneity between studies, where larger values indicate increasing heterogeneity.

## Results

Thirteen studies fulfilled our inclusion criteria and were included in subsequent analysis. This produced a pooled dataset of 796 patients undergoing BCS following bariatric surgery. Characteristics of included studies are in shown in Table 1. Eight of these were prospective observational studies, four were cross-sectional studies and one was a retrospective cohort study. BCS procedures performed included dog-ear correction, abdominoplasty, panniculectomy, dermolipectomy, liposuction, brachioplasty, mammoplasty, breast reduction and thigh lift. The scores used to assess QOL in each study are outlined in Table 1. The length of follow-up ranged from 2 to 42 months.

**Table 1:** Body contouring studies reporting on changes in QOL and/or psychosocial function after surgical intervention in post-bariatric patients

Author	Year	Design	Quality score	Metabolic operation	BCS operation	Total participants	Follow-up (months)	Instrument used to assess QOL/psychosocial function
Van der Beek [15]	2010	Retrospective	4	RYGBP, LAGB	Abdominoplasty, dermolipectomy, dog-ear correction, liposuction, breast augmentation/reduction	43	42	OPSQ
De Zwaan [11]	2014	Cross-sectional	5	RYGBP, SG, LAGB	Abdominoplasty, thigh lift, breast lift, brachioplasty	314	> 12	MBSRQ, IWQOL, GAD-7, PHQ-9
Koller [23]	2013	Prospective	3	RYGBP, LAGB	Lower trunk lift	27	6	FBeK, WHOQOL-Bref
Modarressi [17]	2013	Prospective	7	RYGBP	Abdominoplasty, mammoplasty, cruroplasty, brachioplasty	98	26	HRQOL
Singh [24]	2012	Cross-sectional	6	RYGBP	NS	46	NS	SF36
Azin [25]	2014	Cross-sectional	4	RYGBP	NS	58	NS	SF36, GAD-7, PHQ-9
Coriddi [18]	2011	Prospective	2	NS	Abdominoplasty, panniculectomy, lower body lift	49	NS	Adapted Barthel ADL and FRI
Bolton [26]	2003	Prospective	3	NS	Abdominoplasty	37	2	RSES, FNE, BESAQ, MBSRQ
Menderes [27]	2003	Prospective	3	VBG	Abdominoplasty, mammoplasty, thigh lift, liposuction, gynecomastia	11	NS	DAS-59, GSC
Song [13]	2006	Prospective	4	NS	Panniculectomy, abdominoplasty, breast reduction, brachioplasty	18	3–6	HRQOL, PBSQOL, Beck’s, BISA, CIBA
Pecori [14]	2007	Cross-sectional	5	BPD	Mastoplasty, abdominoplasty, leg/arm lift, torsoplasty	20	24	BUT
Stuerz [28]	2008	Prospective	6	LAGB	Abdominoplasty	34	12	Strauss and Appelt’s questionnaire, HADS, Life satisfaction questionnaire
Song [29]	2016	Prospective	3	RYGBP	Abdominoplasty, mastopexy, lower body lift, thigh lift, upper arm lift	41	12	MBSRQ, SF-36

*RYGB* Roux-en-Y gastric bypass, *VBG* vertical banded gastroplasty, *BPD* biliopancreatic diversion, *SG* sleeve gastrectomy, *LAGB* laparoscopic adjustable gastric banding, *NS* not specified

### BMI After Initial Bariatric Surgery

Five studies reported on the change in BMI following bariatric surgery only. Pooled analysis demonstrated a weighted mean decrease in BMI of 14 points (− 14.816, 95% CI [− 17.0, − 12.6]), with moderately high heterogeneity (*I*^2^ = 77.8%).

### BMI After Body Contouring Surgery

**Fig. 2:**
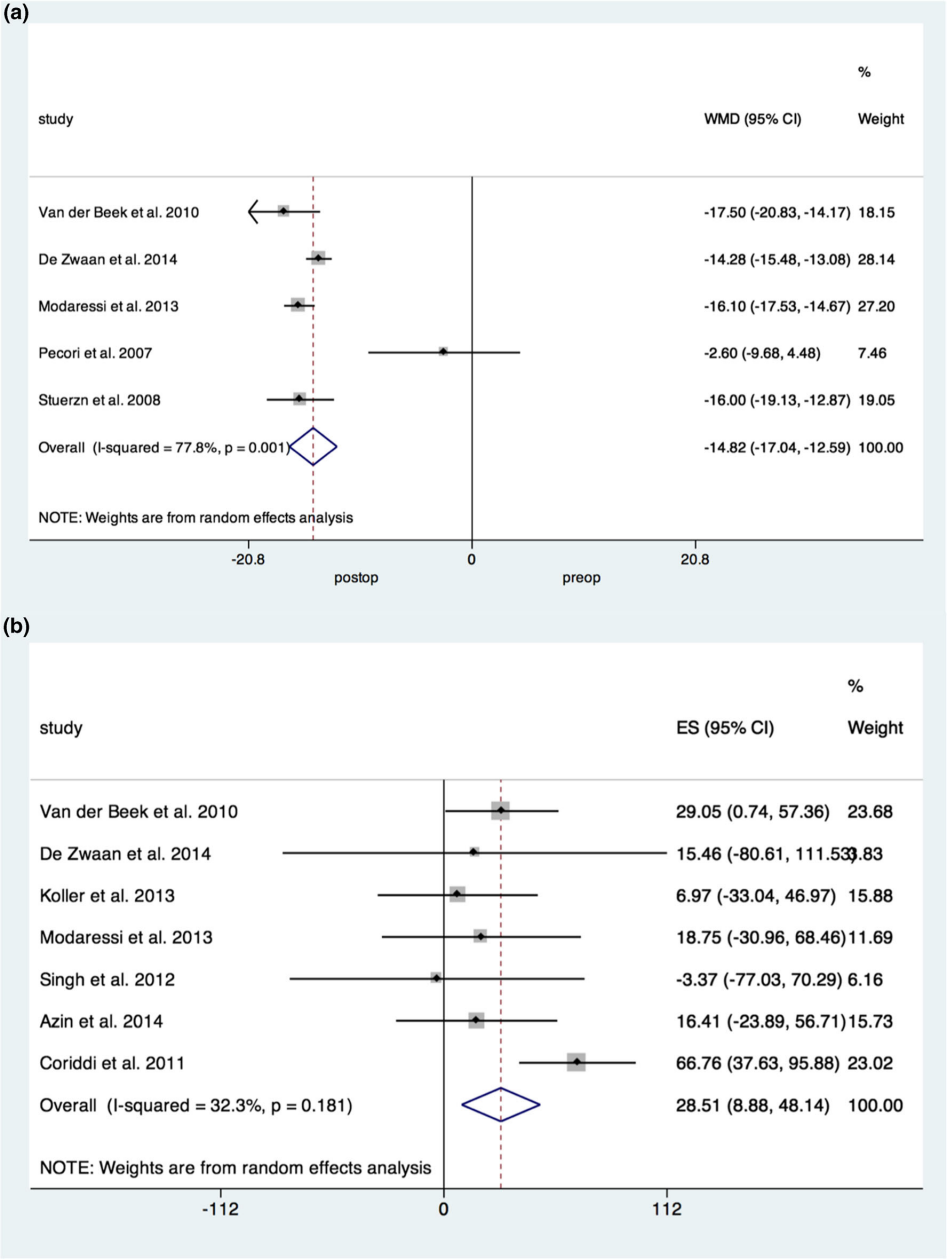
Forrest plots demonstrating **a** a reduction in BMI following body contouring surgery and **b** increase in physical functioning following body contouring surgery

Three studies reported on the change in BMI following BCS. Pooled analyses revealed a significant weighted mean decrease in BMI of 2 points (− 1.99, 95% CI [− 2.99, −0.98]), with no heterogeneity present (Fig. 2a).

### Physical Functioning

Seven studies reported on changes in physical functioning following BCS. Pooled analysis demonstrated a significant improvement in physical functioning by 28.5% (*p* = 0.004, 95% CI [8.9, 48.1]), with a low degree of heterogeneity (*I*^2^ = 32%) (Fig. 2b).

### Psychological Wellbeing

Six studies reported on changes in psychological wellbeing following BCS. Pooled analysis demonstrated a significant improvement in psychological wellbeing by 45.7% (*p* = 0.029, 95% CI [4.7, 86.7]); however, a high degree of heterogeneity was present (*I*^2^ = 87%).

### Social Functioning

Eight studies reported on improvements in social functioning after BCS. Pooled analysis revealed a significant improvement in social functioning by 24% (*p* = 0.001, 95% CI [10.0, 38.0]), with a low degree of heterogeneity (*I*^2^ = 29%).

### Body Image

Eight studies assessed for improvements in perception of body image following BCS. Pooled analysis demonstrated an improvement in body image by 55%; however, this result was not significant (*p* = 0.12, 95% CI [− 14.3, 125.6]).

### Sexual Function

Five studies observed changes in sexual functioning after BCS. Pooled analysis demonstrated an improvement in sexual functioning by 49.7%; however, this result was not significant (*p* = 0.238, 95% CI [− 32.8, 132.1]), and heterogeneity was high (*I*^2^ = 97%).

### Pain

Three studies assessed for improvements in pain following BCS. Pooled analysis demonstrated a non-significant improvement in pain by 18.5%, with high heterogeneity (*p* = 0.4, 95%CI [− 26.7, 63.6], *I*^2^ = 82.9%).

### Self-Esteem

Three studies reported on changes in self-esteem following BCS. Analysis revealed a non-significant improvement in self-esteem by 17.6% (*p* = 0.4, 95% CI [− 27.8, 63]).

### Global QOL Score

Five studies reported a change in the overall QOL using a global QOL score that included a range of physical and psychosocial outcomes. One of these studies used the SF-36 score, two studies used the Health-Related Quality of Life (HRQOL) score, one study used the Impact of Weight on Quality of Life Questionnaire (IWQOL) score and one study used the World Health Organization Quality of Life (WHOQOL) score. Pooled analysis demonstrated an improvement in overall QOL by 14.2% (*p* = 0.083, 95% CI [− 1.9, 30.2]).

### Quality Scoring

All included studies were assessed for their methodological quality and risk of bias using a modified Newcastle-Ottawa Scale. Five studies were of moderate-high quality. Of these, only one study was scored as high quality (≥ 7) [17]. Eight studies were scored as low quality. All studies met the criteria for ascertainment of treatment and clearly defining outcomes of interest. The majority of studies also met the criteria for adequately reporting follow-up procedures. However, many studies scored poorly on comparability. This may be attributable to the lack of randomisation, which increases vulnerability to selection bias. The methodological quality of included studies is shown in Table 2.

**Table 2:** Methodological quality of included studies as assessed by review authors. Asterisks ≥ 5 represent moderate-higher quality and asterisks < 5 stars represent lower quality

Author	Selection			Comparability	Outcome		Total
	1	2	3	4	5	6	
Van der Beek (2010) [15]	*	–	*	–	*	*	4
De Zwaan (2014) [11]	*	–	–	**	*	*	5
Koller (2013) [23]	*	–	–	–	*	*	3
Modarressi (2013) [17]	*	–	–	****	*	*	7
Singh (2012) [24]	*	–	*	***	*	–	6
Azin (2014) [25]	*	–	*	*	*	–	4
Coriddi (2011) [18]	*	–	–	–	*	–	2
Bolton (2003) [26]	*	–	–	–	*	*	3
Menderes (2003) [27]	*	–	*	–	*	–	3
Song (2006) [13]	*	–	*	–	*	*	4
Pecori (2007) [14]	*	–	–	***	*	–	5
Stuerz (2008) [28]	*	–	–	***	*	*	6
Song (2016) [29]	*	–	–	–	*	*	3

The Cochrane ROBINS-I tool for non-randomised studies was used to assess the risk of bias in all included articles (Table 3). With the exception of two studies, all included articles had a moderate overall risk of bias. Most studies scored poorly on measurement of outcomes. This was primarily due to a lack of patient and assessor blinding, a common caveat in surgical trials [30]. However, measures to overcome this source of bias were unaddressed or unreported by most studies. A degree of performance bias was also present in most studies since peri-operative outcomes are particularly vulnerable to this [31]. Another domain of concern was the risk of confounding. This may be attributable to QOL being our outcome of interest. QOL is multifactorial and subjective in nature; therefore, outcomes associated with QOL are influenced by a multitude of other factors, which were unable to be controlled for. Funnel plot assessment was used to assess the degree of publication bias in included studies. Statistical analysis using Egger’s test did not reveal any significant small-study effects.

**Table 3:**
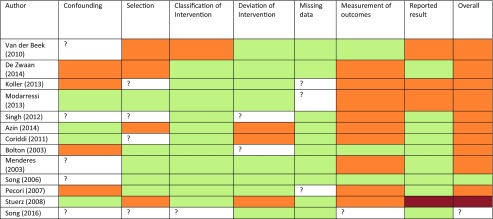
Risk of bias in non-randomised studies (ROBINS-I)—assessment of each risk of bias item according to review authors

means the study is comparable to a well-performed randomised trial

of bias means the study is sound for a non-randomised study with regard to this domain but cannot be considered comparable to a well-performed randomised trial

of bias means the study has some important problems

of bias means the study is too problematic to provide any useful evidence on the effects of intervention

(?) No information on which to base a judgment about risk of bias for this domain

## Discussion

This systematic review and meta-analysis included 13 studies evaluating the effect of BCS on QOL in post-bariatric patients. Overall, our pooled analyses demonstrate that BCS in post-bariatric patients results in statistically significant improvements in numerous indicators of QOL, specifically physical functioning (28.5% increase), psychological wellbeing (45.7% increase) and social functioning (24% increase). These improvements in QOL were also associated with statistically significant, though modest weight loss following BCS. The benefit of BCS on sustaining weight loss was also observed by Froylich [32], who demonstrated post-bariatric patients who had undergone BCS maintained weight loss for a significantly longer period than a matched cohort who did not have BCS.

We also demonstrated improvements in body image, sexual functioning, pain, workability and self-esteem, although these were not significant. The lack of significant effect size in body image has previously been explained [33] where patients tend to demonstrate a discrepancy between their expectations from BCS and the ideal body type they desire. When choosing their ideal body type, participants selected a silhouette one size smaller than the body shape they believed to be attainable from BCS. As a result of this phenomenon, patients would remain dissatisfied with their body image despite the significant weight loss achieved from BCS. This is a common finding in patients with body dysmorphic disorder who present with an extreme preoccupation with an imagined or mild defect in appearance, resulting in significant social, psychological and occupational impairment [34]. As a result, many of these patients seek cosmetic surgery; however, evidence suggests that this cohort still reports a high rate of dissatisfaction with treatment outcomes [35]. Additionally, results revealed that although psychological wellbeing significantly increased, improvements in self-esteem were not significant. It would seem plausible that improvements in mood would be accompanied by similar effects in self-esteem; however, it has been observed that self-esteem and psychological wellbeing acted independently to each other [13].

Although the pooled analysis of global QOL scores revealed an improvement following BCS, this was not a significant result. This is surprising given that individual domains of QOL such as physical, social and psychological functioning demonstrated such significantly positive effects. This finding may be attributable to the wide variation in global scores used among included studies to assess overall QOL. For example, SF-36, HRQOL, WHOQOL and IWQOL were included in the range of global scores used by different studies to quantify the overall changes in QOL following BCS.

It is now established that massive weight loss following bariatric surgery leads to both physical and psychological impairments due to development of loose and ptotic skin, with areas of redundant adipose tissue interfering with activities of daily living [36]. The resulting impact on mobility, body image perceptions and mood prevent post-bariatric patients from fully re-integrating themselves into society [13, 37]. Therefore, weight loss alone may not result in sustained improvements in QOL [23] and further surgical intervention through BCS may offer one route to achieve enhanced lifestyle and psychological goals. Currently, many healthcare providers consider BCS procedures to be predominately cosmetic and merely an adjunct to bariatric surgery [15] and therefore, these are not associated as part of a multimodal treatment to enhance patient quality of life for obesity.

In the UK, current NICE guidance on the management of obesity simply encourages increased information on and access to reconstructive surgery where appropriate. Consequently, the criteria determining eligibility for BCS have traditionally been locally determined and therefore received criticisms of precipitating a postcode lottery [38]. The British Association of Plastic, Reconstructive and Aesthetic Surgeons (BAPRAS) have offered more specific guidance on the inclusion criteria for BCS with clearer referral pathways [36]. These include (i) age over 16 years and (ii.a) starting BMI above 40 kg/m^2^ or above 35 kg/m^2^ with comorbidities and (ii.b) current BMI of less than or equal to 28.0 kg/m2, (ii.c) weight stability of 12 months and (ii.d) significant functional disturbance (both physical and psychological). Furthermore, they have recommended the use of central funding and national registry of outcomes and the use of a national referral document [36].

However, the literature demonstrates that in single-payer health systems such as the UK’s National Health Service, there is a low national uptake of these guidelines with only 7% of Clinical Commissioning Groups implementing the guidance, resulting in continued regional variation of BCS rates [39], despite 70% of patients seeking BCS following massive weight loss even when qualifying for surgery. This could be due to (i) the lack of pooled QOL data and an evidence-based consensus regarding BCS outcomes and indications based on QOL and patient-reported outcome measures (PROMS), (ii) the operative risks of BCS surgery following bariatric surgery and (iii) the financial restraints of some health systems. These factors may contribute to the limited drive for healthcare payers to integrate BCS into the routine bariatric surgical pathway.

## Strengths and Limitations

This study is the first to quantitatively meta-analyse improvements in QOL after BCS in patients who have undergone bariatric surgery. Although previous studies have reviewed the existing literature, they have not extracted QOL data and quantitatively synthesised various QOL scores to produce an overall effect [40, 41]. Additionally, our analyses revealed a low degree of heterogeneity in improvements in physical and social functioning, adding robustness to our results. However, the results presented here should be interpreted in the context of a number of limitations.

Firstly, most studies were inherently limited due to their study design, with no randomised controlled trials being eligible for inclusion in our analysis. Secondly, we did observe a significant degree of heterogeneity in other measures of QOL, particularly psychological wellbeing, body image, sexual function and pain. This may be attributable to the wide variation in scoring systems used across studies to measure each indicator of QOL. Other confounding factors contributing to this heterogeneity include variations in methodology, study designs, sample sizes and follow-up periods.

Thirdly, we classified the various indicators of QOL across all studies into defined groups, including physical functioning, social functioning, psychological wellbeing, body image, pain, self-esteem and global QOL. The integration varying through these combined classifications may be a source or bias in our results.

## Conclusion

In summary, we demonstrate that BCS may improve QOL in patients who have previously undergone bariatric surgery. Statistically significant improvements in physical, social and psychological functioning, as well as benefits in body image, sexual functioning and self-esteem suggest that BCS should not be merely a cosmetic adjunct to bariatric surgery, but has a role in reversing the functional and psychological abnormalities that result from the accumulation of excess skin after massive weight loss. Although there is persistent debate on whether BCS should be an optional or essential addition to bariatric surgery, this study provides further evidence that BCS should be considered as an integral part of the bariatric surgical pathway. The evidence presented in this review acts as further encouragement to increase the uptake of post-bariatric BCS guidelines and therefore increase consistency in the regional provision of BCS across the UK and worldwide. Furthermore, these results could be employed in the routine counseling of patients prior to bariatric surgery as evidence-based information regarding the benefits of BCS. Ultimately however, larger prospective and randomised controlled trials, in addition to cost-effectiveness studies of BCS, are needed within the context of centralised databases and increased multidisciplinary practitioner consensus. Together, these can further our understanding of the effects of BCS on QOL and its role in supporting the multitude of current and future weight loss modalities in the management of obesity and its comorbidities.
